# Finite element modeling in obstetrics and gynecology: advances, applications, and challenges

**DOI:** 10.3389/fmed.2025.1606989

**Published:** 2025-08-29

**Authors:** Taylor Moseley, Ashley J. Hicks, Elizabeth M. Cosgriff-Hernandez, Manuel Karl Rausch, Julie Hakim

**Affiliations:** ^1^School of Medicine, Baylor College of Medicine, Houston, TX, United States; ^2^Department of Biomedical Engineering, The University of Texas at Austin, Austin, TX, United States; ^3^Department of Aerospace Engineering and Engineering Mechanics, The University of Texas at Austin, Austin, TX, United States; ^4^Department of Mechanical Engineering, The University of Texas at Austin, Austin, TX, United States; ^5^Department of Obstetrics and Gynecology, Division of Pediatric and Adolescent Gynecology, Baylor College of Medicine, Houston, TX, United States; ^6^Department of Surgery, Division of Pediatric Surgery, Texas Children’s Hospital, Houston, TX, United States

**Keywords:** pelvic floor, computational modeling, incontinence, pelvic floor dysfunction, vagina, uterus, labor

## Abstract

Finite element modeling (FEM) is a critical tool in biomechanics and biomedical engineering, offering valuable insights where *in vivo* or *ex vivo* investigations are not possible. This review specifically highlights the diverse applications of FEM within obstetrics and gynecology through a comprehensive analysis of the literature. We explore the past use of FEM in analyzing complications affecting pelvic floor structures, urinary continence, and reproduction. The potential contributions of FEM in addressing these challenges are summarized and future directions for its application in obstetrics and gynecology are highlighted.

## 1 Introduction

Finite element modeling (FEM) is a powerful numeric simulation tool that can solve complex mechanical problems. That is, it can predict stress and strain in a deformable body in response to internal and external forces. It is important to note that accurately modeling the stress–strain response of soft tissues is challenging due to their complex composition; failure to account for characteristics such as viscoelastic behavior and anisotropy may compromise the accuracy of finite element model predictions. FEM predicts stress and strain by solving the balance of linear momentum equation, which governs the deformation of solids, including the soft and hard tissues (1, 2). In contrast to other numerical approaches, it is applicable even for arbitrarily complex geometries and material behaviors. Therefore, FEM is uniquely well suited for applications in biomechanics in general and soft tissue biomechanics in particular (2). Fundamentally, FEM breaks down geometries into so-called “finite elements,” thereby reducing a generally unsolvable differential equation into a system of solvable, algebraic equations (1). FEM is especially useful where *in vitro* or *in vivo* experimentation is not possible, unethical, or too expensive. In those scenarios, it can provide insight into the underlying mechanics of tissues, organs, and device-organ interfaces that is otherwise inaccessible. Its inverse counterpart, inverse finite element analysis (iFEA), is also informative, focusing on the estimation of material parameters. Using known variables, it applies an algorithmic and iterative approach to determine material constants for various constitutive models (3).

FEM is an indispensable tool in engineering disciplines, used in modeling of continuum mechanics and macroscopic material behavior, including biomechanics and biomedical engineering. Its use has contributed significant insights to fields like cardiology (4), orthopedics (5), and dentistry (6)—particularly in addressing pathologies that are dictated by complex biomechanical phenomena. Over the past 20 years, FEM has been adopted to understand similar functions within obstetrics and gynecology, thereby deepening our understanding of conditions impacting pelvic floor health, ranging from pelvic floor dysfunction (PFD) to obstetric complications. In doing so, FEM has addressed the unique mechanical stressors affecting the pelvic floor, where forces arise from gravitational load, are amplified during physiologic events like childbirth, and accumulate through repeated increases in intra-abdominal pressure during activities such as coughing or lifting. These stressors are further compounded by age-related changes in tissue structure, including collagen degradation and hormonal shifts associated with menopause.

Conditions impacting the pelvic floor and pregnancy have devastating consequences for women. Specifically, PFD impacts around 25% of women (7), with an estimated cost of 1.5 billion dollars for surgical repair of prolapse alone in the United States (8). Unfortunately, damage sustained during labor and delivery leads to pelvic floor dysfunction in up to 10% of women following childbirth (9). Additionally, as many as 26% of women suffer from urinary incontinence following childbirth (10). During pregnancy, cervical insufficiency is a significant cause of preterm birth and may be responsible for up to 20% of second trimester losses (11). The field of gynecology has made progress in identifying risk factors (10, 12, 13) and imaging techniques (14, 15) to assess pelvic pathologies. However, these approaches have not yet identified clear mechanisms behind many of these conditions, which has limited efforts to identify targeted solutions for them. The use of FEM may offer an additional understanding of the mechanical environments surrounding these disorders, which combined with other methods may enable more effective treatments. By integrating patient-specific data, FEM can help explore the pathophysiology and potential treatments of pelvic disorders. It allows researchers to manipulate specific biomechanical variables, such as tissue elasticity, loading conditions, or anatomical structures, and observe how these changes influence mechanical stress, strain distributions, and organ displacement. This capability has contributed to answering key questions about the magnitude and location of strains required to produce symptoms in various pelvic pathologies. While reviews have addressed FEM applications in either gynecology (16–19) or obstetrics (20, 21) this review systematically examines both fields to highlight methodological advances, synthesize insights into key pathologies, and illustrate FEM’s broad and evolving role in women’s health.

## 2 Methods

**Figure 1:**
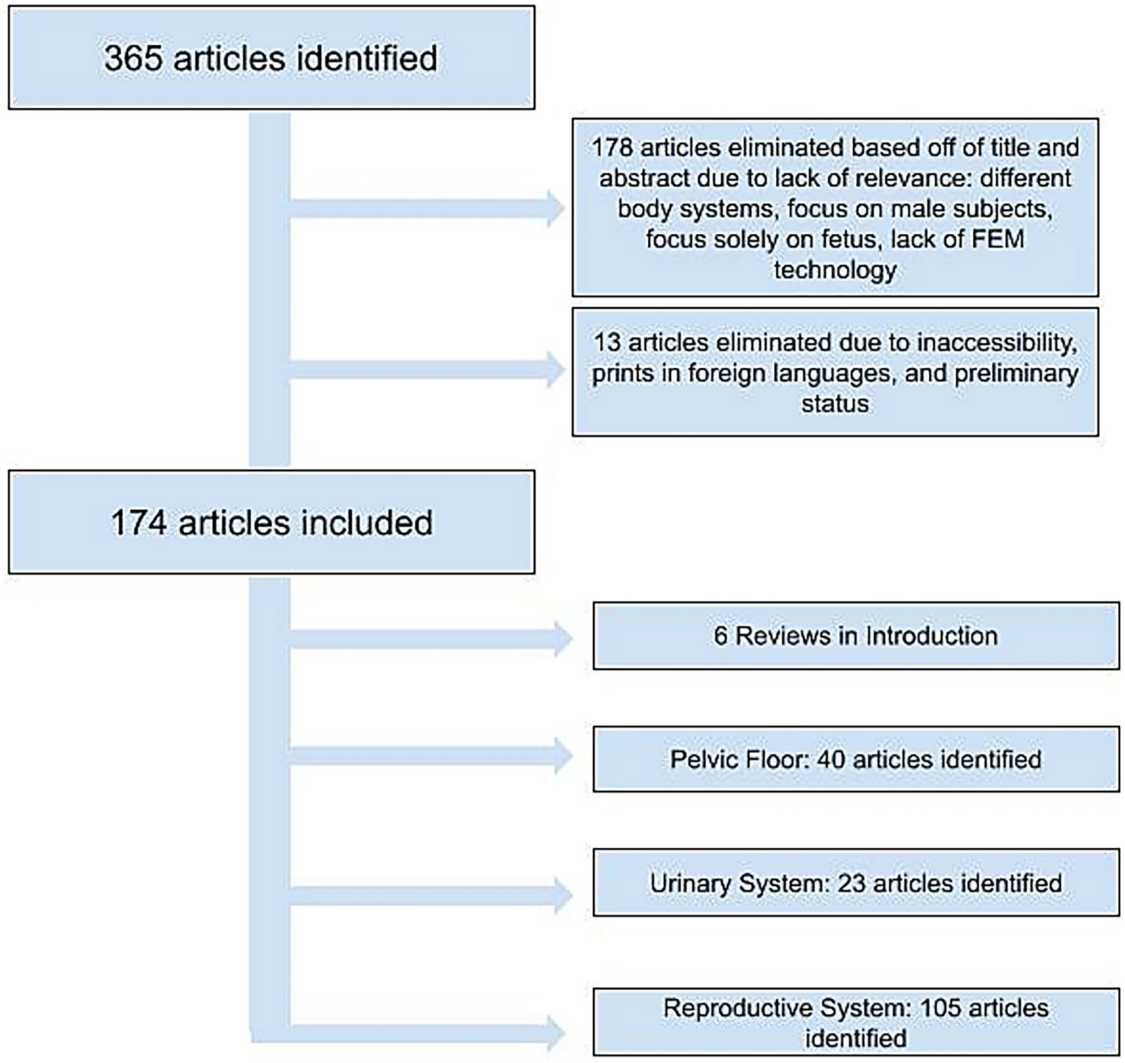
Flowchart of article selection process following PubMed search.

We performed a literature search in pubmed using the terms “finite element” and (“gynecology” or “gynaecology” or “vagina” or “uterus” or “cervix” or “obstetric” or “pelvic floor” or “incontinence” or “urethra”) on 6/19/24. This resulted in 365 articles. 178 were eliminated due to lack of relevance, meaning they focused on non-gynecological body systems, focused solely on the fetus, or lacked finite element modeling. Of the remaining 187 articles, 13 were eliminated due to language, preprint status or inaccessibility (Figure 1). Each of the included articles was grouped by subject area (pelvic floor, urinary system, or reproductive system). Then each subject area was reviewed to identify FEM related contributions.

## 3 Pelvic floor

### 3.1 Anatomy and tissue modeling

**Figure 2:**
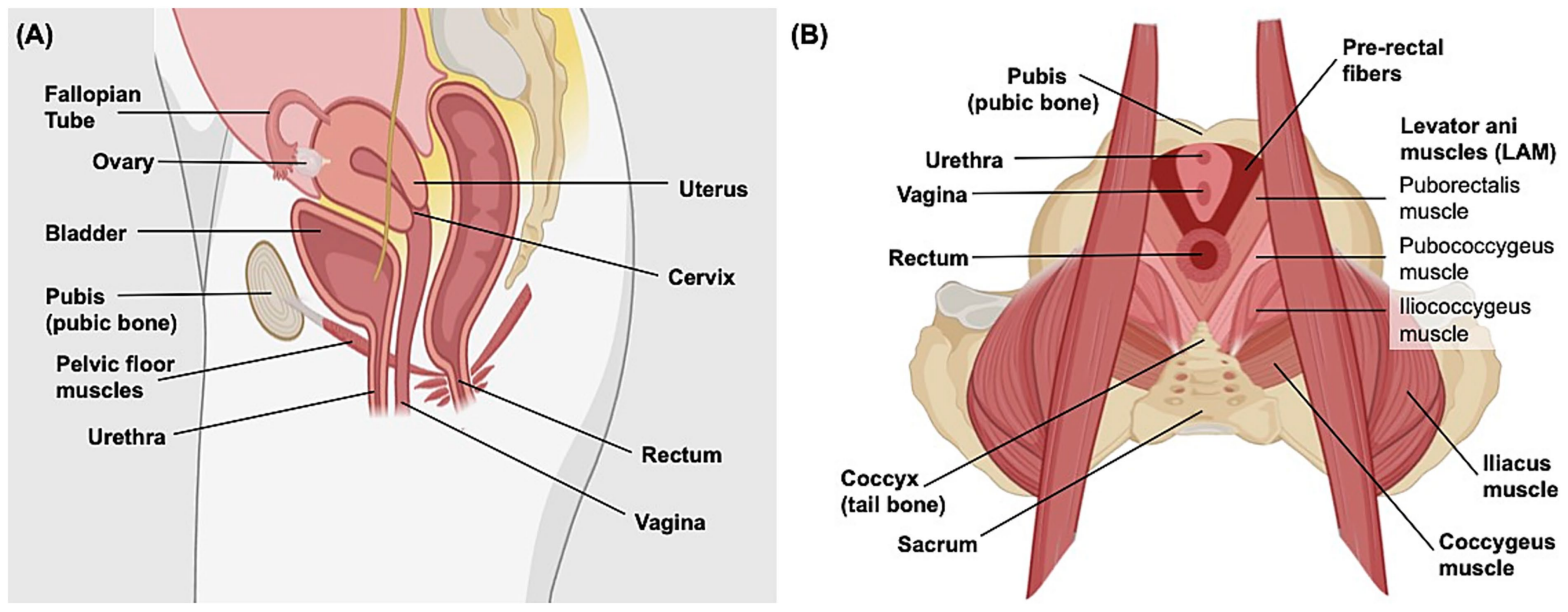
Anatomy of the human female pelvis. **(A)** Sagittal view of the female abdomen demonstrating the general position and orientation of the bladder, rectum, and reproductive organs. **(B)** Superior transverse view of the organization of female pelvic floor muscles, including the levator ani muscle group that is primarily involved in urination, defecation, sexual activity, and supporting pelvic organs. Created in Biorender. (195).

FEM has enhanced our understanding of the pelvic floor’s structural system, which suspends and supports organs essential to reproduction and urination (22). Figure 2 illustrates the anatomical organization of the pelvic floor. We identified 40 articles in this topic area. Initial work created geometric meshes of the pelvic floor using data from magnetic resonance (MR) imaging, computed tomography (CT), and cadaveric analysis (23–25). To simulate the biomechanical behavior of the pelvic floor structures, researchers developed constitutive models that characterize the soft tissue mechanics. Several models incorporated hyperelastic behavior into musculature and ligaments (26–29), with some employing hyperelastic material laws, such as Mooney–Rivlin formulations, to describe the nonlinear behavior of these structures (28, 29). Other work acknowledged the hyperelastic nature of fascia but implemented simplified linear elastic properties in the finite element model (30). Simulations have continued to employ additional, informative modeling techniques. For example, to help estimate the material properties of the pelvic floor, iFEA has been used as a key tool by refining material constants through deformation-based experiments (31). To characterize the behavior of pelvic fascia, one study applied mixture theory, using Voigt’s isostrain model to represent the fascia’s composition of collagen-elastin fibers, adipose tissue, and smooth muscle (32). The development of pelvic floor models have enabled the simulation of normal physiological movements that occur in the pelvic floor. For example, Noakes et al. modeled the Valsalva maneuver (i.e., forcibly expiring against a closed airway leading to increased intrathoracic and intraabdominal pressure (33)) from live patient data to improve the general understanding of levator ani muscle (LAM) group function during this process, using FEM to solve the proposed governing equations of finite elasticity (34). These models paved the way for the adoption of FEM to study gynecologic and obstetric challenges.

### 3.2 Pathology, injury and pelvic organ prolapse

The pelvic floor not only suspends organs against the force of gravity but also supports the primary function of preventing pelvic organ prolapse (POP). Unfortunately, stressors such as obesity, age, LAM injury, and parity (35) cause critical lapses in tissue integrity and compromise this role, leading to pelvic organ herniation. Historically, risk factors for POP were ascertained through correlational studies of women in which prolapse had occurred (35). However, use of FEM has allowed many groups to simulate the effect of potential pelvic floor injuries and determine how the severity and location of these injuries may manifest into POP at different areas within the pelvis, which could not be performed clinically. Newer FEM methodologies introduced anatomically accurate 3D models based on MRI (36, 37), incorporating detailed structures such as the vaginal lumen (38), pelvic floor muscles, and ligaments such as the cardinal and uterosacral ligaments. Xu et al. used a different modality, the Chinese Visible Human (CVH) dataset, to construct their model and further augment anatomical accuracy. The CVH dataset is an anatomical dataset derived from ultra-thin cadaveric sections (39). Through each of these strategies, simulations improved the fidelity of anatomical attachments and boundary conditions. As shown by Mayeur et al., such improvements may significantly enhance the accuracy of pelvic floor displacement models—even more so than variations in soft tissue material properties (40).

In addition, models simulated increased intra-abdominal pressure and revealed potential sites of vulnerability within the pelvis, including ligaments at connection points (36, 38) and the upper anterior vaginal wall (36, 37) in anterior vaginal wall prolapse. Chanda et al. similarly identified stress concentration along the anterior vaginal wall (AVW) when simulating bladder filling using FEM, employing an innovative forced thermal expansion technique to examine the effects of vaginal tissue stiffening in cases of pelvic organ prolapse (41). Their findings revealed that while bladder filling alone led to a relatively uniform stress distribution along the AVW, increasing vaginal tissue stiffness resulted in a growing zone of concentrated stress, particularly at the mid-vagina. Notably, the peak stress levels remained relatively unchanged despite the enlargement of this high-stress zone. This suggested that as prolapse progresses and vaginal tissue stiffens, discomfort may intensify due to the increasing area of strain, even if conventional clinical assessments fail to detect significant changes in stress at the AVW. These results highlight the value of subject-specific computational modeling in identifying subtle biomechanical changes that may not be apparent through standard imaging techniques, potentially aiding clinicians in determining the need for early intervention.

The effects of increased pressures on displacement of pelvic floor-supported organs were assessed by systematically varying: (1) loading conditions, (2) the modeled material properties of the pelvic floor, and (3) the degree of damage to supportive ligaments (Table 1). For example, pairing damage to the anterior vaginal wall with different degrees of intra-abdominal pressure (IAP) identified the amount of pressure needed to produce anterior prolapse (42). Simulations also showed that solely weakening the apical ligaments did not lead to prolapse in several cases (42, 43). Instead, anterior prolapse emerged from specific combinations of apical ligament changes (uterosacral and cardinal ligaments), fascial and muscle damage, and increased IAP (38, 44). By modeling increased IAP and tissue strain across various conditions (Table 1), FEM studies have demonstrated the potential contributions of specific pelvic floor components—such as differences in the levator ani muscle, apical ligaments, and vaginal wall—to the development of organ prolapse (36–38, 41–49).

**Table 1:** Summarizing pelvic organ prolapse simulations: key parameters and results.

Article title		Parameters adjusted	Findings
1	Two-dimensional biomechanical finite element modeling of the pelvic floor and prolapse PMID: 37294482	Simulated different IAPs 50% impairment of USLs CLs and LAM Simulated different utero-vaginal angles	Utero-vaginal angle of 90 degrees with IAP of 148.1 cm H_2_O and impairments led to maximal cervical displacement The combination above created a posterior vaginal wall prolapse, or rectocele
2	3D finite element modeling of pelvic organ prolapse PMID: 27174200	Simulated different IAPs Impaired vaginal wall Simulated impairment of USL and CL	With increased IAP plus vaginal wall damage led to maximal anterior wall prolapse, that did not respond to changes in USL and CL impairment alone
3	Three-dimensional finite element analysis of the pelvic organ prolapse: A parametric biomechanical modeling PMID: 30499117	Simulated different IAPs Simulated ligament impairment of uterosacral, external urethral, pubourethral, and anococcygeal ligaments Simulated muscle impairment of the pubococcygeus (PCM), puborectalis (PRM), and deep transverse perineal (DTP) muscles Simulated muscle activation	IAP and muscle deterioration increasingly affected POP Ligament defect alone did not have a significant impact on prolapse Muscle activation was protective against POP
4	A 3D finite element model of anterior vaginal wall support to evaluate mechanisms underlying cystocele formation PMID: 19481208	Simulated different IAPs Simulated ligament impairment: cardinal and uterosacral ligaments Simulated muscle impairment: levator ani	IAP of 100 cm H_2_O alone created a small cystocele IAP of 168 cm H_2_O plus damage to the listed muscles and ligaments led to the largest cystocele Cystocele was impacted by large apical impairment (20% remaining stiffness) and by LAM impairment LAM impairment led to larger urogenital hiatus
5	A 3D finite element model of uterus support to evaluate mechanisms underlying uterine prolapse formation PMID: 36562389	Simulated different IAPs Simulated ligament impairment: cardinal (CL), uterosacral (USL), broad ligament (BL), and round ligament (RL)	Uterine displacement and ligament stress was sensitive to ligament injury and IAP increase, individually and in combination
6	Three-Dimensional Modeling of the Pelvic Floor Support Systems of Subjects with and without Pelvic Organ Prolapse PMID: 25710033	Simulated different IAPs Simulated anterior vaginal wall and ligament damage (cardinal and uterosacral ligament)	Vaginal displacement was most sensitive to damage to ligaments and vaginal wall
8	A multi-compartment 3-D finite element model of rectocele and its interaction with cystocele PMID: 25757664	Simulated different IAPs Simulated damaged muscle: LAM Simulated damaged ligaments: uterosacral and cardinal Simulated damage to “anterior and posterior support”	Simulated rectocele with the following: damaged LAM, and “posterior support” under increasing IAP– with uterosacral and cardinal ligament damage, this was increased Simulated cystocele with LAM, “anterior support,” “posterior support” and apical support damage Noted that under conditions where cystocele or rectocele may occur, reducing cystocele increased rectocele
9	Relationship between high intra-abdominal pressure and compliance of the pelvic floor support system in women without pelvic organ prolapse: A finite element analysis PMID: 36004379	Simulated different IAPs	Anterior vaginal wall was more sensitive to increased IAP Larger displacement of the vagina was noted at the top
10	Pathophysiological aspects of cystocele with a 3D finite elements model PMID: 27402504	Simulated under pressure of 10 cm H_2_O For assessing medial cystocele: lengthened pubocervical and endopelvic fascia For lateral cystocele: lengthened arcus tendineus fasciae pelvis (ATFP) and arcus tendineus levator ani For apical cystocele: lengthened ligament and cardinal ligament	For median cystocele: pubocervical fascia is the most significant For lateral cystocele: it was particularly sensitive to changes to ATFP For apical cystocele: Noted greater sensitivity to uterosacral cardinal than the influence In each case: Lengthening the suspension system led to larger displacement
11	Development of anatomically based customizable three-dimensional finite-element model of pelvic floor support system: POP-SIM1.0 PMID: 31263537	Simulated different IAPs Adjusted the length of anterior vaginal wall, uterosacral and cardinal ligaments, and paravaginal fascia Simulated increased hiatus size and LAM avulsion	The pelvic floor was sensitive to each parameter tested, but notably sensitive to LAM avulsion, resulting in maximal displacement
12	A biofidelic computational model of the female pelvic system to understand effect of bladder fill and progressive vaginal tissue stiffening due to prolapse on anterior vaginal wall PMID: 26732347	Simulated bladder filling Simulated different degrees of vaginal tissue stiffness	Increased vaginal tissue stiffness increased the distribution of stress against the anterior vaginal wall with a 50% full bladder
13	A finite element analysis of different postures and intra-abdominal pressures for the uterine ligaments in maintaining the normal position of uterus PMID: 36977774	Simulated different IAPs Simulated different body postures: used different tilts of the upper body Assessed displacement of USL, CL, BL and RL	Increased IAP resulted in increased displacement: uterine movement toward the rectum, cervical movement toward the vagina, and overall downward displacement For posture: leaning back led to more displacement than leaning forward or standing upright USL, CL and RL experienced greater displacement when leaning back BL experienced greater displacement when leaning forward

IAP, Intraabdominal pressure; USL, Uterosacral ligament; CL, Cardinal ligament; LAM, Levator ani muscle; PCM, Pubococcygeus muscle; PRM, Puborectalis muscle; DTP, Deep transverse perineal muscle; POP, Pelvic organ prolapse; BL, Broad ligament; RL, Round ligament; ATFP, Arcus tendineus fasciae pelvis.

Interestingly, FEM also revealed a slight paradoxical protection of cystocele against rectocele and vice versa by assessing the impacts of one form of prolapse upon another (45, 50). FEM demonstrated that the presence of either a cystocele or rectocele restricted the other by showing that when one was removed from simulation, the other grew slightly more pronounced (45).

#### 3.2.1 The levator ani muscle role in prolapse

Within the pelvic floor, the levator ani muscles (LAM) have been modeled extensively using FEM and examined with iFEA. The LAM are a group of muscles that form a hammock-like support of the pelvic floor, serving as the limiting factor to preventing POP through the hiatal opening (51). To better understand the impact of LAM damage on pelvic floor function, Silva et al. developed and analyzed comparative models of both healthy patients and those with pelvic floor pathologies, including urinary incontinence and POP (52, 53). Using iFEA, they assessed the pubovisceralis muscle and found higher material parameters in hyperelastic constitutive models derived from patients with POP, indicating increased tissue stiffness, along with greater force generation. These differences were hypothesized to result from changes in muscle fiber size and collagen content (52). Such findings highlight the utility of iFEA in identifying patient-specific material alterations associated with pelvic floor dysfunction.

The LAM’s passive (34, 52, 54, 55) and active (29, 52, 56) movements during activities such as the Valsalva maneuver or contracting the pelvic floor have also been explored with FEM and iFEA. Demonstrating similar results to imaging studies (57), the LAM hiatus was observed to reduce under active contraction. With FEM, the impact of these contractions under increased pressure could be compared to a pelvis at rest. Higher pressures increased the hiatal opening; however, activation of the LAM reduced these changes (26, 58, 59). Using FEM to simulate LAM damage validated the significance of this muscle group. Specifically, under increased IAP, models with LAM damage had larger hiatal openings (46, 47), avulsion (46), and more significant prolapse (43, 45–47). Adding apical ligament damage to the model caused significant posterior vaginal wall strain, resulting in a rectocele (37, 45). For both POP and LAM impairment, FEM revealed that damage to specific supportive ligaments results in an adverse, additive effect—a finding that could not have been evaluated experimentally.

### 3.3 Surgical and device interventions

FEM has also been used to analyze medical interventions aimed at treating and reducing prolapse. A few studies have modeled surgical mesh repair and assessed the resultant reduction in displacement of the vagina (60, 61). FEM also helped evaluate the optimal mesh structure suture type (single vs. continuous) (61), mesh porosity (62), and suture number (63) to minimize movement and stress. With regards to suture type, the mesh was anchored at discrete nodes to represent the distinct fixation points of the simple stitch. For the continuous stitch, anchoring occurred across a continuous line of nodes, akin to running sutures. They found that while both the simple stitch and continuous stitch improved displacement, the continuous stitch reduced the supero-inferior movement of the uterus and vaginal wall (61), and an increased number of stitches did not produce reduced mobility (63). Similarly, FEM also assessed the efficacy of sacrospinous fixation and the optimal anchorage location to reduce pelvic organ displacement (64). Lastly, FEM was used to improve non-surgical options such as modeling underwear supporting the bladder neck (65) and ways to reduce movement of vaginal pessaries to make them more comfortable (66).

## 4 Urinary system

### 4.1 Modeling urinary mechanics and continence

The mechanisms behind urinary incontinence have also been explored using FEM. We identified 23 articles on the application of FEM to the urinary system. In addition to developing bladder filling and displacement models (67–71), simulations have been used to demonstrate the impact of increased IAP on the bladder and urethra (72) as well as coordination and support from structures within the urinary system (73) and surrounding pelvic floor. By strategically altering the location and constitutive properties of urinary organs, FEM has been used to identify interconnected functions of the urinary tract and supporting structures, including the vascular plexus (74), levator ani (32, 75, 76), perineal membrane, and surrounding pelvic tissue (76, 77). For example, simulating urinary tract structures revealed that the urethra does not open independently at normal detrusor pressures. Instead, the inferior motion that occurs with LAM contraction enables urethra opening (75). The relationship between continence and LAM activity was also supported by pathological models using FEM and iFEA. For instance, modeling urinary tract structures based on women with incontinence and those with continence produced differences in the displacement (53) and angle of the LAM during Valsalva (32). Finally, by incorporating fluid–structure interaction (FSI)—a modeling method that assesses the interplay between fluids and solids—the model by Attari et al. was able to evaluate the urethral vascular plexus, surrounding structures and continence in greater detail. This coupling allowed the simulation to capture how changes in blood and urine pressures deformed the tissue. A reduction in urethra muscle stiffness in combination with altered vasculature impacted urethral sphincter closure pressure by compromising its ability to maintain a seal, and reducing urethral length (74), potentially impacting continence. Table 2 highlights several urinary continence mechanisms identified through FEM studies, including the roles of striated muscle contraction, vascular support, ligament integrity, and connective tissue behavior.

**Table 2:** Summary of finite element modeling studies on urinary continence mechanisms.

Title (with PMID)	Dimensionality and approach	Imaging modality and modeling details	Findings
On Structure-Function Relationships in the Female Human Urethra: A Finite Element Model Approach PMID: 33782810	3D and 2D Axisymmetric Finite Element Model	Derived from MRI; Used 3D multiphysics (Mooney Rivlin hyperelastic) for tissue; 2D fluid–structure interaction with Navier–Stokes for fluid domains	Striated muscle contraction most effectively increased urethral closure pressure. Vascular plexus aids closure. Age-related muscle atrophy greatly reduces closure pressure.
Urethral support in female urinary continence part 2: a computational, biomechanical analysis of Valsalva PMID: 33787951	3D Finite Element Model	Derived from MRI; Soft tissues were modeled as linear elastic solids using Young’s modulus and Poisson’s ratio	Introduced the “Swing Theory.” Found that weakening of perineal membrane or urethral softening alone can produce stress urinary incontinence-like motion.
A computational analysis of the effect of supporting organs on predicted vesical pressure in stress urinary incontinence PMID: 32152891	2D Axisymmetric Finite Element Model	Derived from urodynamic test data; Used fluid–structure interaction methods: urine was modeled as a Eulerian fluid using equation of state and bladder and urethra were modeled as Lagranian solids	Supporting organs increase bladder pressure during stress events. Hyperelastic materials improved prediction accuracy.
Assessment of urethral support using MRI-derived computational modeling of the female pelvis PMID: 26224383	3D Finite Element Model	Derived from MRI; Urine modeled as an elastic liquid and linear elastic solid for soft tissue	Vaginal wall and pelvic muscles (puborectalis, pubococcygeus) are critical for urethral support. Urethral movement was linearly related to abdominal pressure.
A finite element model validates an external mechanism for opening the urethral tube prior to micturition in the female PMID: 25326768	3D Finite Element Model	Derived from X-ray, EMG data, and anatomical literature; Used hyperelastic material properties for soft tissues	Detrusor pressure alone was insufficient for urethral opening; supports active, muscle-driven mechanism.
Modelling of Soft Connective Tissues to Investigate Female Pelvic Floor Dysfunctions PMID: 29568322	3D Finite Element Model	Derived from cadaver data (E12 sheet plastination); Used hyperelastic materials and mixture theory for fascia composition	Weakened fascia caused more urethral hypermobility than ligament damage. Supports the idea that fasciae are critical for continence.
Biomechanical study on the bladder neck and urethral positions: simulation of impairment of the pelvic ligaments PMID: 25527889	3D Finite Element Model	Derived from MRI; Used Ogden and Yeoh hyperelastic models for soft tissue	Impairment of pubourethral ligaments caused significant changes in bladder neck and urethral position. Simulation results matched clinical MRI data.
An estimation of the biomechanical properties of the continent and incontinent woman bladder via inverse finite element analysis PMID: 38523483	Inverse Finite Element Analysis	Derived from MRI; Used Ogden hyperelastic constitutive model for soft tissues	Incontinent women had 47% stiffer bladder tissues and required higher abdominal pressure for similar bladder deformation.
Pressure Transmission Theory—The Rasputin of Incontinence PMID: 35535753	2D Finite Element Biomechanical and Anatomical Model	Derived from X-ray and ultrasound; Used biomechanical vector modeling (integral theory)	Pressure Transmission Theory (PTT) invalidated by 11 distinct methods; Integral Theory-supported midurethral sling shown to restore continence by reinforcing pubourethral ligament without bladder neck elevation. Finite Element Modeling showed pressure transmission insufficient to close urethra.

Summary: Finite Element Modeling studies consistently show that effective urinary continence in women relies on the integrity of pelvic floor muscles, connective tissues (like fascia and ligaments), and their coordinated mechanical function. Striated muscle contraction, especially from the pelvic floor, is critical for urethral closure, while weakened fascia or ligament damage leads to increased urethral mobility and stress incontinence. Age-related atrophy and stiffer bladder tissues further compromise continence. Overall, structural integrity and muscular reinforcement contribute significantly to urethral function.

Outside of the urethra, simulating weakening of the LAM, paraurethral and pubourethral ligaments and supporting fascia showed that these vulnerabilities may produce urethral and bladder neck deformation during straining conditions such as increased IAP or during the Valsalva maneuver (32, 76–78). Silva et al. utilized both FEM and iFEA to investigate biomechanical differences in bladder function between continent and incontinent women. Through iFEA, they identified higher Ogden hyperelastic material parameters in incontinent women, indicating increased bladder tissue stiffness. FEM simulations, supported by MRI data, revealed greater bladder neck displacement in these women, highlighting both tissue-level and structural contributors to stress urinary incontinence (79). Finally, FEM also was utilized to model and evaluate several interventions to address urinary incontinence, including laser treatments (80, 81), single incision slings (82), MiniSlings (83), spinal cord epidural stimulation (84), and midurethral slings (85, 86).

### 4.2 Athletic activity and urinary continence

FEM was also utilized to understand the role of athletic activity in urinary continence. Comparing continent female athletes to incontinent female athletes, FEM revealed no significant difference in the displacement of the pelvic floor muscles during pelvic floor contraction, despite thicker pubovisceral muscles in incontinent women (87). FEM also simulated the urinary tract during a jump to assess which variables (height of jump, fullness of bladder) produced the most urinary leakage (88), as well as the precise malformations to the bladder throughout the activity (89). Two studies noted deformation within the pelvis during jump landings, involving both the pelvic bones (88) and the bladder itself (89). Interestingly, neither identified levator ani muscle (LAM) weakness as a primary cause of incontinence during this activity. Instead, both studies emphasized dynamic factors—specifically, asymmetric deformation between the anterior and posterior pelvis (88) and significant increases in IAP (89)—as key contributors to stress urinary incontinence.

## 5 Reproductive system

### 5.1 Reproductive tissue characterization

In addition to addressing gynecologic concerns, FEM served as a valuable tool to investigate challenges impacting reproductive organs and their respective functions. We identified 105 articles in this area. Similarly to the pelvic floor structures, mechanical experiments established and refined tissue parameters of the cervix, vagina (90, 91), and uterus (92–94). FEM and iFEA were also applied at the cellular and tissue level to characterize abnormal mechanical and biophysical properties in pathological tissues, such as those found in cancer (95, 96), endometriosis (97), and cysts such as cortical inclusion cysts in the ovary (98). FEM and iFEA have been also extensively used to ascertain cervical tissue properties, including electrical impedance (99–104) hydration (105), compressibility (106), and tissue stiffness (106–115)—measurements that could be used to characterize cervical changes during pregnancy. Furthermore, some studies have used FEM to demonstrate the process of pregnancy-related cervical swelling (116), integrating several of these properties in its analysis. Understanding these properties may be particularly useful in assessing cervical insufficiency, a condition with no clear etiology.

To investigate potential maladaptive conditions contributing to cervical strain, FEM was used to simulate different anatomical and material parameters, including IAP, cervical length, tissue scarring, and fiber orientation (117–119). These models revealed that under pressure akin to that experienced during labor contractions, areas of high strain were located at the inner os of the cervix (117, 118). By manipulating cervical length, an established risk factor for cervical insufficiency, Westervelt et al. identified specific changes to the strain patterns of the cervical os. Interestingly, shortening the cervical length from 4 to 2.5 cm in the model caused changes in both stress and strain—but only when simulating softer cervical tissue (117). In fact, softer tissue alone led to increased tissue strain within the cervix, and this effect was further amplified when combined with other factors such as cervical length. When simulating an incompetent cervix (<2.5 cm), stress was distributed more broadly across the cervix, extending beyond the internal os, unlike in models with normal cervical lengths, where stress was more concentrated near the internal os (118). This suggests that a shorter cervix experiences a more widespread mechanical load, potentially contributing to its increased susceptibility to deformation.

Using FEM, tissue structures within the pelvic floor were manipulated in ways that would be impossible *in vivo*. Specifically, altering the alignment of the uterocervical canal to the uterus (117) affirmed the benefit of proper cervical placement; tilting the cervix posteriorly increased the stretch at the cervical os. Also, adjusting the adhesion and thickness of fetal membranes (117) demonstrated that fetal membranes reduce strain on the uterus by redistributing the load of the fetus. Therefore, having thicker and more adherent tissue resulted in less uterine strain.

### 5.2 Fertility and conception models

FEM-based simulation has also greatly advanced our understanding of obstetrics from conception to contractions to delivery. Early studies demonstrated the applicability of FEM in simulating the uterus and adnexa (120). Over time, FEM has been adapted to model various morphological changes in the fallopian tubes (121) and sperm (122), as well as to assess the impact of those changes on fertility. Models captured age-related changes in the tubes, including reduced tubal diameter and cilia (121). In addition, they examined various components of sperm quality, adjusting the size of sperm tails and heads (121, 122). Such models were novel in capturing a range of sperm morphologies and tubal parameters, and in their ability to predict fertility success. Furthermore, by simulating aging reproductive tracts (122) and adjusting sperm motility and number (121), FEM demonstrated the potential to create personalized fertility models that could optimize reproductive outcomes.

### 5.3 Pregnancy and labor simulations

Following conception, FEM and iFEA have advanced existing pregnancy models—for example by identifying constitutive parameters of the uterus (92, 93, 123, 124) and placenta (125, 126) and simulating a pregnant uterus at-term (194). These advancements have enhanced progress toward creating physiologically accurate models. Beyond the uterus, FEM has also been applied to explore the mechanical behavior of the pelvic floor during labor (128–130). For instance, Li et al. developed a subject-specific model of the pelvic floor to evaluate how different constitutive laws affect predictions of childbirth mechanics. They found that using a nonlinear exponential model, as opposed to a neo-Hookean one, led to significantly higher predicted delivery forces and altered stretch distributions in the LAM, highlighting the need for high-strain material data (129). In a related simulation, they also examined the role of mechanical anisotropy in the LAM and showed that increased fiber-direction stiffness (relative to cross-fiber stiffness) reduced the required delivery force and decreased peak muscle stretch (130). Parente et al. explored parameters; they demonstrated how variations in material parameters—even within the same constitutive model—substantially affected predicted pelvic floor strains, emphasizing FEM sensitivity to parameter choice (131).

FEM also progressed our understanding of the strains, forces, and injuries that occur during childbirth. Table 3 outlines key variables explored in these models—including labor duration, fetal size and position, and maternal pushing strategies—highlighting their influence on pelvic floor stress and injury. One such study, by Lepage et al., modeled localized strain on the uterosacral ligaments during delivery and found significant deformation—around 30%—was observed, with important implications for pelvic organ prolapse (132). As expected, multiple models identified significant stress on the perineum (133–137) and LAM during labor (127, 129, 135, 138–143), with several simulations localizing stress to the puborectalis (138, 141, 142, 144), pubococcygeal muscles (139, 142, 143, 145) and attachment sites to the bone (145–147). However, through FEM, researchers were able to assess the interplay between these structures. As models simulated the loosening (134) and removal (135) of the perineum, they demonstrated a decrease in LAM stress during labor, affirming the perineum as a significant source of tension on the LAM.

**Table 3:** Biomechanical factors influencing pelvic floor stress and injury during labor and delivery.

Category	Articles involved	Findings
Labor duration	On the effect of labour durations using an anisotropic visco-hyperelastic damage approach to simulate vaginal deliveries (PMID: 30170191)	30170191: Precipitous labor increased labor forces and pubovisceral damage; resting phases allowed tissue recovery.
	Viscous effects in pelvic floor muscles during childbirth: A numerical study (PMID: 28886617)	28886617: Precipitous labor caused highest tissue stress; prolonged labor led to lower peak stress due to viscoelastic relaxation.
	A biomechanical study of the birth position: a natural struggle between mother and fetus (PMID: 35384526)	35384526: Occipito-anterior position resulted in faster fetal descent (by ~4 min)
Pushing pattern (differences in length of push and rest)	On the effect of labour durations using an anisotropic visco-hyperelastic damage approach to simulate vaginal deliveries (PMID: 30170191)	30170191: Delayed pushing reduced muscle force (~7%) and damage (~3%).
	On the management of maternal pushing during the second stage of labor: a biomechanical study considering passive tissue fatigue damage accumulation (PMID: 35101408)	35101408: More pushes per contraction (5 vs. 3) increased fatigue-related damage; shorter pushes (5 s vs. 10 s) also resulted in less damage.
	The influence of an occipito-posterior malposition on the biomechanical behavior of the pelvic floor (PMID: 19272693)	19272693: Pushing in occipito-posterior position caused greater stretch (1.73 vs. 1.63) in pelvic floor muscles compared to occipito-anterior position- particularly impacting the levator ani and pubococcygeus muscles
Perineal protection	The role of thumb and index finger placement in manual perineal protection (PMID: 24842121)	24842121: Manual perineal protection reduced perineal stress, especially with correct finger placement during crowning.
	Modeling manual perineal protection during vaginal delivery (PMID: 23835811)	23835811: Finger support lowered perineal tension; minimal benefit from poor technique.
	Fetal head size and effect of manual perineal protection (PMID: 29287104)	29287104: Adjusting finger position optimized perineal tension reduction; the proper technique was particularly effective in reducing the elevated tension associated with larger fetal head sizes.
Maternal birthing position	Effect of the birthing position on its evolution from a biomechanical point of view (PMID: 33422852)	33422852: Flexible sacrum positions (e.g., squatting) reduced fetal head reaction forces (175 N vs. 239 N), allowed greater coccyx rotation (15.7° vs. 3.6°), and required less pubic symphysis widening (3 mm vs. 6 mm), though they caused slightly higher pelvic floor muscle (PFM) stress
Fetal dynamics		
Fetal head molding	Study on the influence of the fetus head molding on the biomechanical behavior of the pelvic floor muscles, during vaginal delivery (PMID: 25757665)	25757665: A deformable fetal head reduced stretch and reaction forces on the pelvic floor. Maximum stretch ratio dropped from 1.532 (rigid) to 1.504 (deformable).
Fetal head size	Biomechanical pregnant pelvic system model and numerical simulation of childbirth: impact of delivery on the uterosacral ligaments, preliminary results (PMID: 25227746)	25227746: Larger fetal head size during childbirth increases deformation of the uterosacral ligaments—raising the risk of pelvic support damage
	Simulation of the Childbirth Process in LS-DYNA (PMID: 38299474)	38299474: Increasing head diameter from 80 mm to 90 mm raised pelvic floor muscle stress by 11–27% and slowed delivery progression.
Fetal head position	The influence of an occipito-posterior malposition on the biomechanical behavior of the pelvic floor (PMID: 19272693)	19272693: The occipito-posterior position caused greater pelvic floor muscle stretch (1.73 vs. 1.63) than occipito-anterior.
	A biomechanical study of the birth position: a natural struggle between mother and fetus (PMID: 35384526)	35384526: The occipito-posterior position led to more pelvic floor stress, joint stress, slower descent and more extreme coccyx rotation.
	Persistent occiput posterior position and stress distribution in levator ani muscle during vaginal delivery computed by a finite element model (PMID: 31197428)	31197428: The occipito-posterior position increased puborectalis stress by 3.6×.

Summary: Biomechanical modeling studies highlight several key factors that influence pelvic floor stress and injury during labor. Short, intense labors and improper pushing patterns can elevate tissue damage, while controlled pushing and appropriate rest phases reduce stress. Manual perineal protection—especially with correct technique—effectively minimizes perineal tension, particularly in cases with larger fetal heads. Flexible birthing positions and fetal head molding also play a protective role by reducing reaction forces and tissue stretch. Conversely, occipito-posterior fetal positions consistently increase stress and deformation of pelvic structures.

FEM has been useful in advancing our understanding of how fetal position, head size, and pelvic anatomy interact to influence maternal tissue strain during childbirth. Investigations focused on fetal positioning, comparing occiput anterior (OA) and occiput posterior (OP) presentations during descent (139, 140, 148). The OP position was found to place greater strain on the LAM, yielding higher maximal principal stresses. FEM allowed for a more detailed characterization of OP-related stress, fostering analysis of stress distribution across different fetal head descent stations during labor (140) and revealing its potential impact on fetal head molding (148). Beyond positioning, FEM was applied to assess the effect of fetal head size on maternal tissue strain. Several studies reported that larger fetal head diameters correlated with increased stress on the pelvic floor muscles (145, 149, 150). Notably, Yan et al. further developed a partial least squares regression model to predict key deformation metrics during childbirth (149). Meanwhile, Tao and Grimm found that uterine stress was minimally affected by head size, in contrast to the fetal neck and pelvic floor, where stress increased (150). Finally, FEM was applied to evolutionary questions regarding pelvic design. Although the human pelvis is not optimized in all respects, increasing its size or altering its anterior shape was found to impair delivery mechanics and reduce overall mobility (151, 152).

FEM models have advanced significantly in simulating fetal dynamics during labor, including fetal head molding and the cardinal movements—flexion, internal rotation, extension, and external rotation—during vaginal delivery (153). These advancements enhanced our understanding of strain patterns on maternal tissues. For example, a 3D FEM model was used to evaluate varying degrees of fetal head flexion. The results showed that greater flexion was associated with reduced pelvic floor stress and a shift in peak stress to lower stations within the birth canal (154). In another study, simulations of fetal head molding demonstrated a notable reduction in pelvic floor muscle strain and stretch, with the most pronounced effect observed in the levator ani muscle during vertical descent (155).

Incorporating visco-hyperelastic material properties into FEM models has added significant value by accounting for the time-dependent behavior of tissues under stress. This approach enables a more accurate representation of how strain accumulates over time. One study used this method to evaluate the effects of different maternal pushing patterns on pelvic floor strain, comparing 1, 3, and 5 pushes per contraction, with push durations of 5 and 10 s. The results showed that the least tissue damage occurred with three 5-s pushes per contraction, while the most damage resulted from five 5-s pushes. The study also found that the most damage was sustained during active pushing efforts, rather than being solely related to the total duration of labor (156). However, it is important to note, excessively rapid labor is not ideal for minimizing strain. Precipitous labor—defined as delivery within 3 h of regular contractions (157)—was associated with higher reaction forces compared to normal or prolonged labor (138, 158). Furthermore, brief rest intervals between contractions were shown to increase mechanical strain on pelvic tissues, while longer rests helped reduce maximal reaction force (158, 159).

In addition to identifying factors that increase stress on the pelvic floor, FEM was used to examine other mechanisms that reduced stress and stretch. For instance, FEM revealed that organized fiber orientation in scar tissue minimized stress (160). Birthing positions were also assessed; simulations of different maternal birthing positions found that kneeling or squatting enhanced the flexibility of the coccyx, reduced widening of the pubic symphysis, and alleviated strain on the pubic ligaments (161). Additionally, finite element simulations showed that external rotation of the femurs—by inducing lateral expansion of the ilia and increasing tension in the ilio-sacro-transverse and axial ligaments—leads to a measurable enlargement of the pelvic inlet area. This mechanism may be clinically useful in situations where increased inlet space is beneficial, such as in cases of obstructed labor or shoulder dystocia (162). Athletic status did not appear to have a large impact on stretch, but athletic women appeared to produce more force in one simulation (147). From a clinical perspective, simulations have also reinforced actionable insights for physicians by demonstrating the protective effects of manual perineal support—identifying optimal finger placements to reduce high-strain areas in the perineum (163, 164) and confirming this method’s utility across a range of fetal head sizes (165). Finally, FEM models optimized the angle and length of episiotomies to reduce strain, particularly in the more strenuous occiput posterior (OP) fetal positioning (166–169).

### 5.4 Modeling uterine contractions

Finite element modeling of uterine contractions has progressed significantly through the incorporation of greater physiological detail and structural complexity, enabling more accurate simulation of uterine stress and function. FEM models have been significantly refined by integrating detailed physiological mechanisms, including uterine smooth muscle cell electrical activity, intracellular calcium dynamics, myosin phosphorylation, and filament sliding (170). Enhancements have allowed for more realistic simulations of contractile behavior by incorporating both active contractile elements and passive tissue properties (150, 171). Additionally, the anatomical accuracy of the models was improved by expanding the fiber architecture of the uterine wall to include three simulated fiber directions: longitudinal, circumferential, and normal (150, 172). Using magnetomyography data, FEM was also employed to compute the lead-field matrix that maps uterine electrical activity to the magnetic fields detected by sensors, in order to better understand patterns of uterine activity (173). Finally, FEM also facilitated evaluation of how different contraction patterns affect uterine stress. Notably, simulations revealed that tachysystole elevated stress levels during relaxation, while shortened resting intervals between contractions resulted in the highest overall stress levels (159).

### 5.5 Device testing and clinical applications

FEM was used to improve technology that could benefit from non-invasive methods, such as pregnancy-related trauma, training, and device testing. After developing a novel balloon dilator to expand the cervix, Filipovic et al. employed FEM to evaluate differences in strain on the cervix non-invasively (174). Similarly, Asiedu et al. applied FEM to assess the safety of competing designs and materials for a new, pen-sized colposcope before human use (175). FEM was also used to improve the safety of therapies, including hyperthermic treatments for cancer (176–178), endometrial ablation (179, 180), as well as assessing the impacts of the heat produced by transvaginal transducers (181). To support training and simulation, FEM was used to help identify the ideal material for physical training models (182). FEM also helped lay the groundwork for creating hysteroscopy (183–185), childbirth (186) and gynecological surgical simulations. Finally, FEM has been used to simulate dangerous situations, including the responses of pregnant women and fetuses in road accidents (187–192) and military blasts (193), enabling the analysis of tissue and fluid responses to trauma as well as fetal injury.

## 6 Summary and future directions

FEM is a powerful tool with wide-ranging applications across the field of obstetrics and gynecology. FEM has facilitated the recreation and manipulation of complex pelvic structures to better characterize the biomechanics underlying pathologies of the pelvic floor, pregnancy, and childbirth. Although informative, it is important to note that FEM simulations have limitations.

First, many simulations lacked validation against imaging studies or experimental data. In several cases, this was due to ethical and technical constraints. For example, *in vivo* assessment of tissue damage during pregnancy or labor was limited by concerns for patient comfort and safety. As demonstrated in Table 4, which focused on validation of pelvic organ prolapse simulations, validation methods varied considerably. While several studies incorporated mesh convergence, sensitivity analysis, or qualitative comparison to clinical trends, only a few performed quantitative validation using MRI-based displacement or statistical assessments. Several models had no direct validation attempts. This variability highlights the ongoing challenge of achieving both anatomical fidelity and experimental validation.

**Table 4:** Validation approaches in finite element models of the pelvic organ prolapse models.

Article title	Attempt at validation?	Brief validation summary
Finite element studies of the deformation of the pelvic floor	No	No experimental or subject-specific validation nor comparison to imaging data.
A shell finite element model of the pelvic floor muscles	No	No experimental or subject-specific validation nor comparison to imaging data.
Pathophysiological aspects of cystocele with a 3D finite elements model	No	No experimental or subject-specific validation nor comparison to imaging data.
A biofidelic computational model of the female pelvic system to understand effect of bladder fill and progressive vaginal tissue stiffening due to prolapse on anterior vaginal wall	No	No experimental or subject-specific validation nor comparison to imaging data.
Three-dimensional finite element analysis of the pelvic organ prolapse: A parametric biomechanical modeling	Minimal	Limited quantitative comparison of select model outputs to values reported in previous literature; no direct experimental or in vivo validation conducted.
Effect of material properties on predicted vesical pressure during a cough in a simplified computational model of the bladder and urethra	Partial	Compared simulated vesical pressure to urodynamic data; noted limitations of using vesical pressure alone for validation, as it is not sensitive to variations in model parameters
Three-dimensional modeling of the pelvic floor support systems of subjects with and without pelvic organ prolapse	Partial	Compared trends and structures with clinical observations; no quantitative nor experimental validation conducted
Two-dimensional biomechanical finite element modeling of the pelvic floor and prolapse	Partial	Compared trends and structures with clinical observations; no quantitative nor experimental validation conducted
A multi-compartment 3-D finite element model of rectocele and its interaction with cystocele	Partial	Compared trends and structures with clinical observations, photos and MRI; no quantitative nor experimental validation conducted
3D finite element model of anterior vaginal wall support to evaluate mechanisms underlying cystocele formation	Partial	Compared predicted anterior vaginal wall deformations under abdominal pressure to dynamic MRI images; no quantitative nor experimental validation conducted
Development of anatomically based customizable three-dimensional finite-element model of pelvic floor support system: POP-SIM1.0	Partial	Compared model outputs with clinical exams and stress MRI images; no quantitative validation conducted
Vaginal Changes Due to Varying Degrees of Rectocele Prolapse: A Computational Study.	Yes	Compared rectocele size results to clinical data from a published cohort study of patients diagnosed using ultrasound imaging
Physical-based statistical shape modeling of the levator ani	Yes	Performed perineometer validation, MRI-based shape comparison, and principal component analysis to distinguish pathological cases
Biomechanical properties of the pelvic floor muscles of continent and incontinent women using an inverse finite element analysis	Yes	Quantitatively compared simulated displacements to MRI data; used optimization to minimize error
3D finite element modeling of pelvic organ prolapse	Yes	Simulated in vivo cervix traction experiment; matched clinical IAP thresholds
Relationship between high intra-abdominal pressure and compliance of the pelvic floor support system in women without pelvic organ prolapse: A finite element analysis.	Yes	Compared simulated displacements to dynamic MRI and literature values
Modeling the contraction of the pelvic floor muscles	Yes	Compared displacements with MRI data from the same subject with quantitative assessment
Subject specific finite elasticity simulations of the pelvic floor	Yes	Compared simulated to MRI displacement; vertical error ~2.6%, horizontal error ~74.5%
Characterizing the Biomechanical Properties of the Pubovisceralis Muscle Using a Genetic Algorithm and the Finite Element Method	Yes	MRI-based displacement validation; <17% error
An approach on determining the displacements of the pelvic floor during voluntary contraction using numerical simulation and MRI	Yes	Compared numerical displacements with MRI data from the same subject; relative error between the model’s 100% contraction output and the MRI measurement was 2.8
Constriction of the levator hiatus during instruction of pelvic floor or transversus abdominis contraction: a 4D ultrasound study	Yes	4D Ultrasound validated, repeatability tested, statistical significance confirmed
Characterization of the passive and active material parameters of the pubovisceralis muscle using an inverse numerical method. J Biomech	Yes	Compared simulated displacement to MRI; error <3%; cylindrical model verification

This table categorizes published FE modeling studies of the pelvic organ prolapse models based on the extent of validation attempted: studies labeled as “no validation” lacked any experimental, subject-specific, or imaging-based comparison; those with “minimal validation” compared only a few model outputs to values reported in the literature without direct clinical, imaging, or experimental confirmation; “partial validation” involved qualitative comparisons to clinical observations, prior literature, or limited imaging data; and studies marked as “yes” included quantitative comparisons using subject-specific data, imaging such as MRI or ultrasound, or in vivo experiments.

Second, FEM is a simulation tool sensitive to its inputs, including boundary conditions, anatomical anchoring points or pressures applied to the model, as well as constitutive assumptions and formulations. For example, in pelvic floor modeling, while some teams chose to model each muscle of the LAM group, others simulated it as a singular entity—an approach that may have impacted the precision of the results. Further, many models of the pelvic floor simplified or excluded structural supports such as ligaments or fascia, reducing the accuracy of boundary constraints, which are inherently influenced by adjacent anatomical supports. In urinary system models, common simplifications included modeling the bladder as a spherical structure or omitting urine flow entirely. In childbirth simulations, several models omitted key aspects of fetal dynamics by modeling only the fetal head while excluding the body, or by representing the head as rigid without accounting for molding Across each of these systems discussed, several studies relied on cadaveric or animal-derived tissue properties, which may not have reflected the mechanical behavior of living human tissue under physiological conditions. Of course, it is important to acknowledge that increasing model complexity and anatomical accuracy often requires greater time and resource investment—striking the right balance between model fidelity and feasibility remains a persistent challenge.

Evidently, there is no established standard for pelvic floor models. Models are oftentimes developed to address a specific need and validated, if at all, using individualized patient data. Therefore, the specificity of these unique models often limits their generalizability.

However, while specificity is a limitation for widespread application, it also aligns with one of FEM’s greatest strengths: its capacity for subject-specific modeling. Using geometric meshes constructed from medical imaging, FEM offers the ability to subject-specific solutions. Future applications of these models could include guiding the design of patient-specific pessaries for pelvic organ prolapse, identifying targeted stretching protocols for individuals with hypertonic pelvic floor dysfunction, optimizing cerclage techniques based on patient-specific biomechanics, and determining ideal positioning strategies for individuals with restricted mobility or those preparing for labor.

Beyond current applications, FEM could create detailed simulations supporting the development of new treatment and diagnostic strategies. Given its ability to model mechanical strain and predict tissue displacement, FEM could be leveraged to assess the risk of ovarian torsion in cases where the ovary is enlarged due to cysts, tumors, or other adnexal masses. By incorporating anatomical data—such as ovary size, vascular pedicle length, mass location, and tissue properties—acquired through medical imaging, FEM can simulate the mechanical forces acting on the adnexa and identify conditions that may predispose to torsion. This predictive approach could aid in clinical risk stratification and guide surgical planning, particularly for patients who are not ideal candidates for immediate or exploratory surgery. With regards to obstetrics, by applying advanced FEM methods (i.e., computational fluid dynamics) to study blood flow during pregnancy, simulations of placental development and vasculature could be generated, which could deepen our understanding of conditions such as placental insufficiency or pre-eclampsia. Once models are created, their use could be extended to develop treatments or even ways to prevent these conditions.

Finally, FEM could help in understanding the impact of pelvic tissue scarring—especially in patients with profibrotic conditions, such as pelvic inflammatory disease (PID), Asherman’s syndrome, or those undergoing oncologic treatments. Models could simulate the fibrotic barriers that hinder ovum transport, and design stents or other devices that optimize the passage of the egg, improving fertility outcomes. Within the vagina, FEM could address post-surgical or radiation vaginal stenosis by aiding in the design of custom degradable stents with the necessary stiffness to interrupt the progression of tissue damage and maximize therapeutic benefit. FEM could accelerate the development of stents, dilators, and other personalized devices by reducing the need for repeated physical prototyping and minimizing dependence on animal models.

In summary, FEM has the potential to significantly enhance our understanding and treatment of conditions in obstetrics and gynecology, ultimately helping to preserve quality of life for patients with pelvic pathologies. To fully harness this potential, future efforts should prioritize model standardization, rigorous validation, and integration into clinical workflows. With continued refinement, FEM holds great promise for transforming diagnostics, uncovering the biomechanics underlying common disorders, and improving patient outcomes across the field.
